# Quantitative Measurement of Rotation in Phalangeal Fracture Malunion Using Computed Tomography Imaging—“Linkage Simulation”

**DOI:** 10.3390/diagnostics14161818

**Published:** 2024-08-21

**Authors:** Hidemasa Yoneda, Katsuyuki Iwatsuki, Masaomi Saeki, Atsuhiko Murayama, Nobunori Takahashi, Michiro Yamamoto, Hitoshi Hirata

**Affiliations:** 1 Department of Human Enhancement and Hand Surgery, Nagoya University, 65 Tsurumai-cho, Showa-ku, Nagoya 466-8560, Japan; 2 Department of Limb Traumatology, Nagoya University, 65 Tsurumai-cho, Showa-ku, Nagoya 466-8560, Japan; 3 Department of Orthopedics, Aichi Medical University, 1-1 Yazakokarimata, Nagakute 480-1195, Japan

**Keywords:** linkage simulation, malunion of phalangeal fracture, malunion of metacarpal fracture, scissoring of the fingers

## Abstract

Malunion of thumb and finger fractures causes problems in the cosmetic and functional aspects of the hand. Malunion of phalangeal fractures usually manifests as a combination of rotational deformities in the coronal, sagittal, and transverse planes, and corrective osteotomy is performed on the planes that cause these problems. Quantification of the deformity is essential for precise osteotomy and is difficult to perform in the transverse plane, even with radiography or computed tomography. Thus, we developed a technique called linkage simulation for the quantitative measurement of rotational deformities for surgical planning. In this procedure, finger extension and flexion can be simulated based on the predicted rotational axis of the joint, which is useful for determining the appropriate correction. Furthermore, by performing a reduction simulation in the software, it is possible to simulate the surgery and predict the postoperative results. This paper reports the details of this technique.

## 1. Introduction

Fractures of the phalanges and metacarpals can easily result in malunion if the reduction position is not maintained until the fracture unites [1]. Malunions usually manifest as a combination of three-dimensional (3D) deformities in the coronal, sagittal, and transverse planes, with rotational deformities in the transverse plane being the most problematic. This deformity can cause scissoring of the fingers, hampering their use [2]. Radial-ulnar deviation in the coronal plane and flexion/extension deformities in the sagittal plane are rarely overlooked. However, rotational deformities in the transverse plane are often overlooked and not identified on imaging examinations [3,4].

Osteotomy is indicated in cases of malunion, in which functional impairment persists after bone union. In preoperative planning, surgeons usually evaluate the degree of deformity. Measuring deformities in the sagittal and coronal planes is easy using radiography, but it is difficult to quantitatively measure rotational deformities, even using computed tomography (CT). Therefore, during surgery, to verify that the malunion was precisely corrected, one surgeon flexed the operated finger and visibly checked its relationship with the other fingers [5]. If a deformity remains, revision maneuvers are required until an ideal reduction is achieved [6,7].

**Figure 1:**
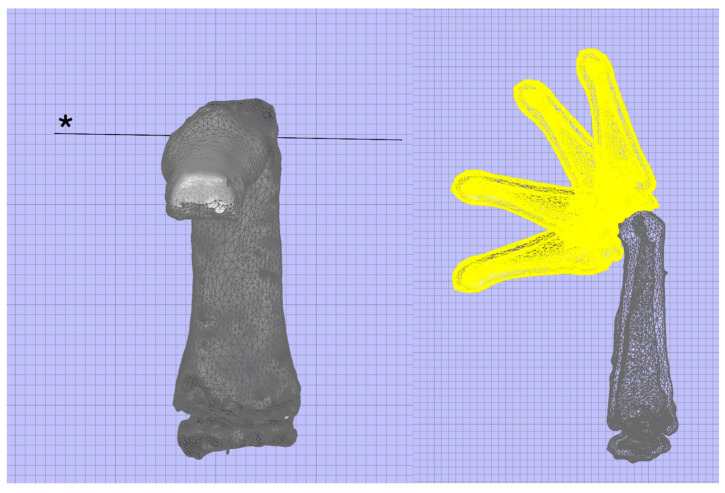
With the linkage simulation, one can estimate the flexion and extension of joints based on the predicted axis of rotation (*).

To estimate the amount of correction needed for the rotational deformity, we previously proposed the “linkage simulation” method, which can estimate the rotational angle of the cross-section using the predicted rotational axis of the joint [8]. In this method, the motion of each joint is simulated by creating a predicted rotation axis. Surgeons can simulate the 3D joint movement of the fingers before and after reduction, which enables prediction of the results of deformity correction without having to repeat reduction and fixation during surgery (Figure 1, Supplementary Materials). By comparing the predicted axis of rotation caused by the deformation with the original axis, it is possible to quantitatively measure the degree of rotational deformation. After publishing our first report 7 years ago, we improved this simulation technique and achieved good results by applying it to multiple patients. Here, we introduce the details of this method and explain its specific applications in surgery.

## 2. Detailed Procedure

### 2.1. Preparation

CT scanning and segmentation were performed by modifying a method previously used for the radius [9,10]. CT scanning (CANON MEDICAL SYSTEMS, Aquilion ONE 64 CT scanner, Otawara, Tochigi, Japan; 80 kV, 100 mA) was performed, and the slice thickness was set to 0.4 mm for segmentation of the finger bones. The finger position for the scan was either extended or flexed without deformities or contractures. Scanning in a flexed position can reduce the number of steps required for the simulation procedure. 

**Figure 2:**
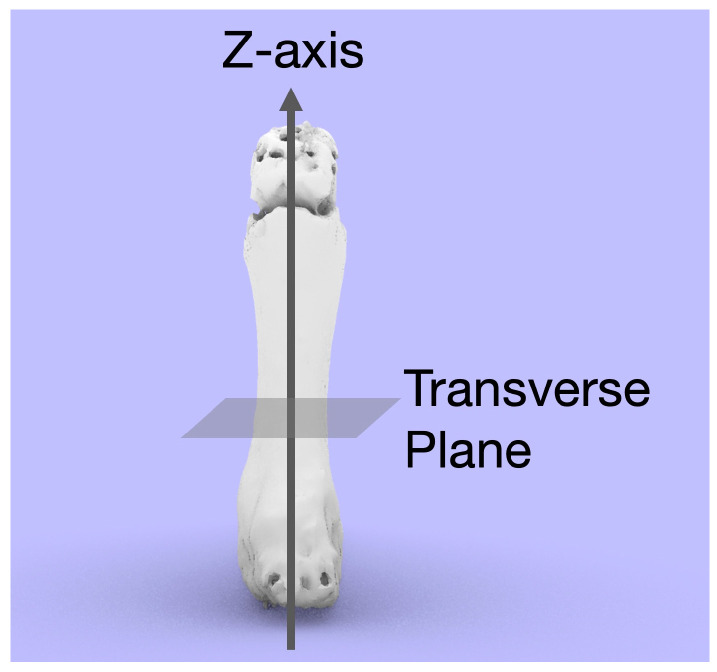
The inertial axis that coincides with the longest axis of the bone is set as the Z-axis, and the plane perpendicular to the axis is defined as the transverse plane.

Next, the bones were segmented using Mimics 21.0 (Materialise, Leuven, Belgium). The created 3D surface model was exported in stereolithography (STL) format and imported into 3-Matic 17.0 (Materialise, Leuven, Belgium). Software such as Rhino 7.0 (Robert McNeel & Associates, Seattle, WA, USA) can also be used. In this software, the correction can be evaluated by moving the finger bones using the Grasshopper plug-in (Robert McNeel & Associates, Seattle, USA) (Figure 1, Movie). The axis of the model was set using the 3D-CAD software. The axis of each bone was determined. The Z-axis represents the long inertial axis of the bone, and the transverse section is perpendicular to it (Figure 2). The method used to determine the X- and Y-axes is described in the following section.

### 2.2. Creating the Predicted Rotational Axis in the Sagittal Plane

Circular approximation was used to create the predicted axis of rotation. Flexion and extension movements of both the PIP and MP joints cannot be achieved by rotation around a single axis but rather involve rotation and translational movement in the sagittal plane [11]. In addition, the metacarpal joint has rotational freedom caused by the morphology of the articular cartilage and laxity of the ligaments, and the rotational axis rotates slightly during flexion [12]. Although such information is essential for an accurate simulation, we defined rotation on a concentric circle for simplification in this study.

**Figure 3:**
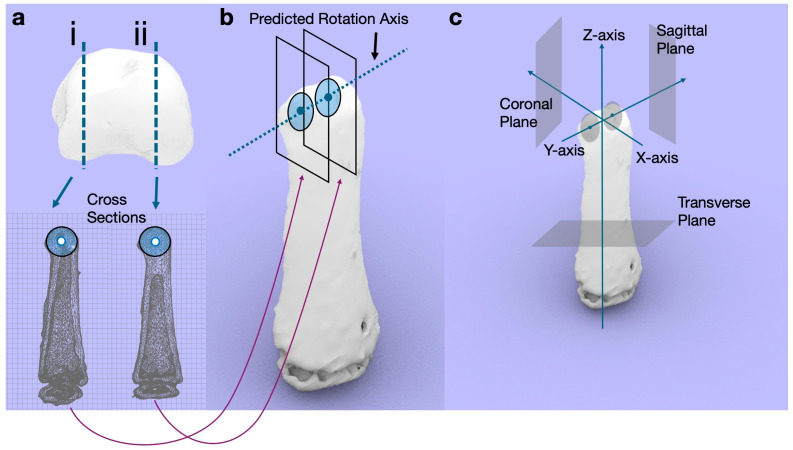
Determination of the predicted rotation axis and the X-, Y-, and Z-axes. (**a**) Circles that fit the shape of the head of the bone at the centers of the radial (i) and ulnar (ii) condyles were drawn. (**b**) The axis connecting the centers of both circles is defined as the predicted rotation axis. (**c**) Next, the predicted rotation axis is defined as the Y-axis, and the plane perpendicular to it is defined as the sagittal plane. The axis perpendicular to the Z- and Y-axes is defined as the X-axis, and the plane perpendicular to the X-axis is defined as the coronal plane.

Two circles were created in the sagittal plane passing through the middle of the radial and ulnar condyles of the bone head (Figure 3). The line connecting the two centers of the circles was defined as the predicted rotation axis. The rotational axis was the Y-axis, and the plane perpendicular to it was the sagittal plane of the bone. The X-axis was perpendicular to the Z- and Y-axes, and the plane perpendicular to the X-axis was the coronal plane specific to the bone (Figure 3).

### 2.3. Planning of the Osteotomy

**Figure 4:**
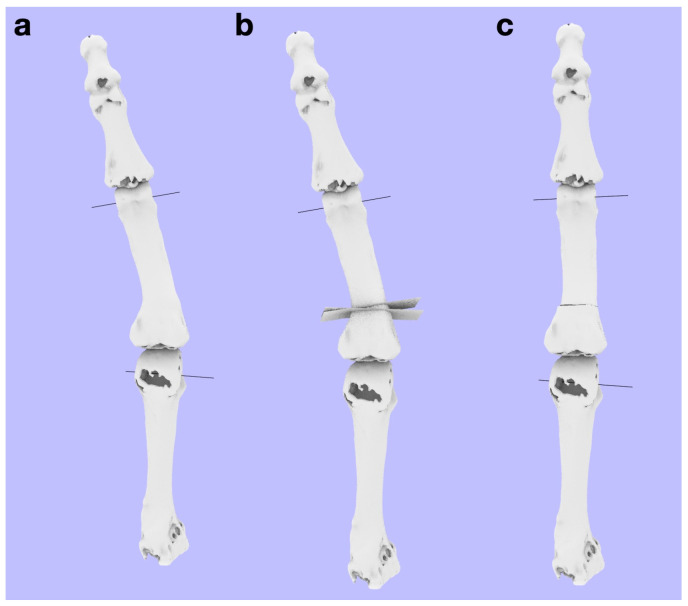
Reduction in the coronal and sagittal planes should be performed before correcting the rotational deformity in the transverse plane. The predicted rotational axis changes when reduction is performed in the coronal plane or the sagittal plane. Then, the axis is defined. (**a**) The proximal phalanx is radially deviated in the coronal plane. (**b**) Therefore, we designed an osteotomy using a closed wedge in the coronal plane. In this case, the proximal osteotomy plane was set perpendicular to the Z-axis. (**c**) After the osteotomy simulation, a predicted axis of rotation was created. Note that the axis differs from the one created before reduction.

The plane of osteotomy was set as a transverse section perpendicular to the Z-axis so that the reduction in the rotation of the malunion can be performed during surgery exactly as that shown in simulation (Figure 4). Repositioning was performed by rotating the bone onto the osteotomy surface. Osteotomies in malunions are usually performed at the fracture sites. However, in many cases, osteotomies are performed at the proximal metaphysis rather than at the site of malunion. This is because moving the osteotomy to the proximal part increases the osteotomy surface area, which provides stability and makes it easier to facilitate union. However, this method is not suitable for correcting deformities in the sagittal or coronal planes.

If reduction in the coronal and sagittal planes was required, an osteotomy was performed at this stage. The decision to perform an open or closed osteotomy is based on the length of the bone [13]. During this procedure, if an open-wedge osteotomy is performed, the cutting surface should be aligned with the transverse section. If a closed-wedge osteotomy is performed, one of the cutting surfaces should be aligned with the transverse section. However, in many cases, the patient’s complaints caused by malunion often disappear with only a rotational reduction in the transverse plane; therefore, it is rare to perform a reduction in the coronal and sagittal planes simultaneously. If a reduction in the coronal or sagittal plane was performed, the axis was defined again. This was because the predicted rotational axis changed. 

### 2.4. Measurement of the Angle of Rotation and Simulation of the Reduction

**Figure 5:**
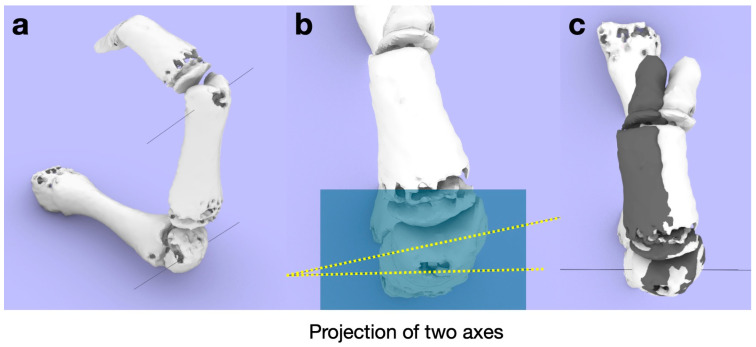
Quantification of rotational deformity in the transverse plane. After the creation of the predicted axes (**a**), the two axes (dotted yellow lines) were projected onto the transverse plane (blue) and the angle between them was measured (**b**). The rotational angle. The distal part of the osteotomy is rotated so that the two axes coincide (**c**).

The bone axes of the proximal and distal joints located at the malunion site were projected onto a transverse section (Figure 5). The angle between the two projected axes was measured as the rotational angle of the transverse plane. The bones distal to the osteotomy site were rotated in the software to simulate reduction such that the projected bone axes coincided.

### 2.5. Confirmation of the Relationship with Other Fingers Using a Linkage Simulation

**Figure 6:**
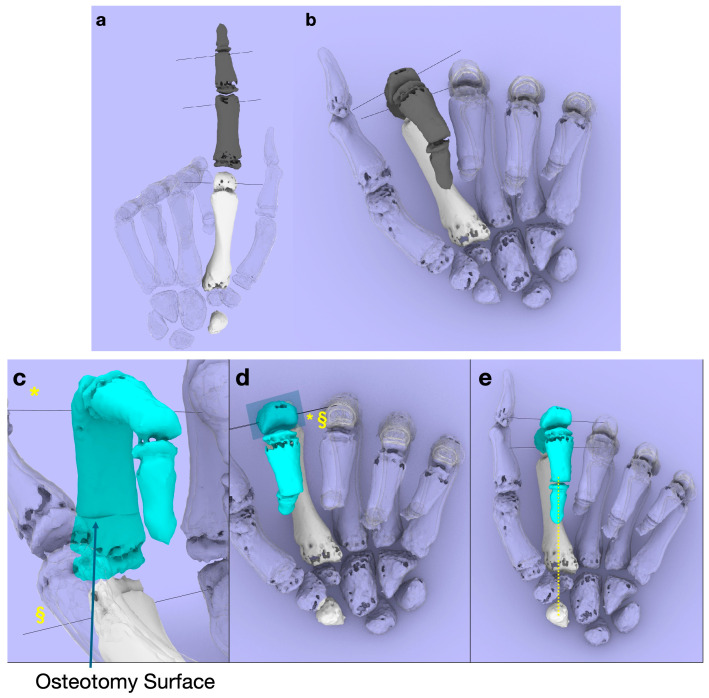
(**a**) Three-dimensional model created from CT images obtained preoperatively. The malunion is noted in the proximal phalanx of the index finger. CT was taken with the index finger extended and middle, ring, and little fingers flexed. The degree of rotational deformity of the transverse section is visually unclear in the extended position. (**b**) Joint flexion using linkage simulation. When all joints are bent, it becomes clear that scissoring is occurring between the index and middle fingers. (**c**,**d**) When the rotation of the transverse section is corrected based on the angles measured following osteotomy, the predicted rotation axes of the PIP and MP joints, which were previously divergent, can be seen to be aligned on the transverse plane. (* indicates the predicted rotation axis of the PIP joint, and § indicates the predicted rotation axis of the MP joint.) In addition, the disappearance of scissoring between the index and middle fingers can be confirmed. (**e**) In the 3D model after reduction, when the MP and PIP joints are flexed and the DIP joint is extended, the inertial axis of the distal phalanx (yellow dotted line) passes through the center of the scaphoid tuberosity.

By flexing the fingers based on each created bone axis, one can check whether scissoring of the fingers has occurred (Figure 6). Rotational reduction can be verified by checking the direction of the nails and finger extensions [14]. Because the fingertips point in the direction of the scaphoid tubercle at maximum flexion [15], it is also possible to check whether the scaphoid tubercle is on the line of extension (Figure 6e). For verification, it is preferable to extend the distal interphalangeal joint rather than flex it.

### 2.6. Application to Actual Surgery

**Figure 7:**
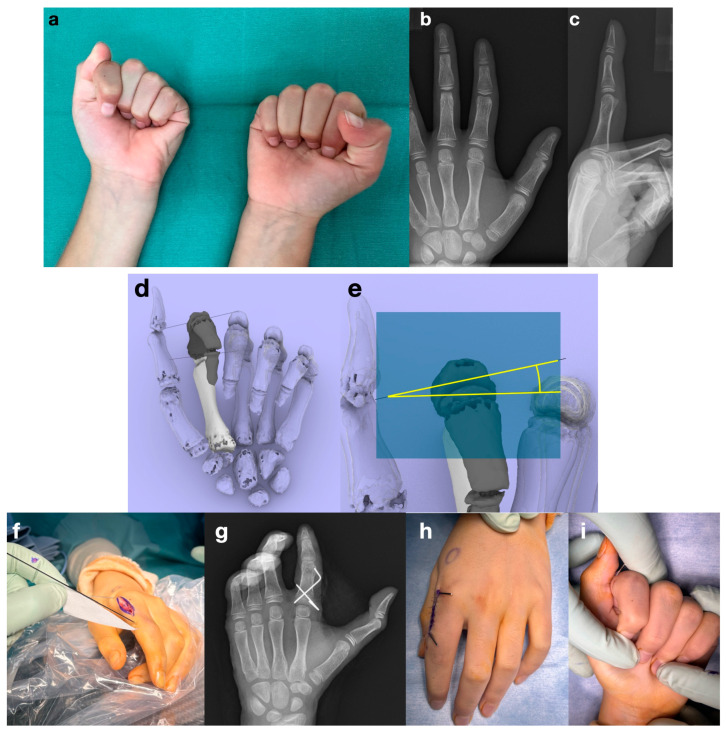
Case presentation with intraoperative photographs. (Case No. 9, (**a**–**c**)) The patient is a 9-year-old boy with a malunion of the metaphysis of the index finger. He had treatment for the fracture when he was 3 years old, but rotational deformity remained and he had scissoring of the left index finger. We considered that the deformity in the coronal and sagittal planes was acceptable, and we decided to correct only the rotation of the transverse plane. (**d**,**e**) With the linkage simulation, we found that scissoring of the index finger could be resolved by supinating the bone fragment by 12 degrees. (**f**–**i**) In the surgery, a template was created to indicate the angle of rotation, and wires were inserted to guide the reduction. Fixation was performed with cross-pinning. The preoperative rotational deformity was resolved after the osteotomy.

The results of the analysis were applied to the surgery group (Figure 7). For the proximal phalanx, the approach is usually performed using a lateral incision to expose the lateral aspect of the phalanx. Before osteotomy, guide pins were inserted into the head of the proximal phalanx and metacarpal so that they were perpendicular to each condyle. After osteotomy, the distal part of the osteotomized area was rotated in the direction in which reduction could be achieved. Cells were fixed using a cross-pinning plate. If a plate is used for fixation, repair of the lateral band can result in its adhesion to the plate. Therefore, the lateral band on the other side was continuous and not necessarily repaired.

We used this technique to treat nine patients based on a previous case report [8] (Table 1). All cases involved treatment of a fracture deformity. The affected sites were the metacarpal bones in four cases and the proximal phalanges in five cases. Four patients required simultaneous reduction in the sagittal and coronal plane, while the deformity in five patients was achieved by correcting only the rotational deformity in the transverse plane.

## 3. Discussion

Malunion after digit fracture is a relatively common complication that affects both the cosmetic and functional aspects of the hand [16]. Deformities in the sagittal plane have little effect on the range of motion if they are small, but if they become more severe, they can cause limitations in the range of flexion and extension [17]. However, even if the degree of coronal plane or rotational deformity is small, finger scissoring can occur [2]. In addition, deformities in the transverse plane are easily overlooked on X-ray scans, and it is rotational deformities that are most likely to be the target of treatment for malunion. To avoid missing the deformity immediately after trauma, it is necessary to flex all fingers and check that there is no deformity. However, if finger flexion is limited because of pain or there is swelling immediately after the trauma, there is a possibility that the rotational deformity can be underestimated.

It is difficult to quantitatively measure the rotational deformation in the transverse plane in malunion, even with CT, and there is a need for a method to quantitatively measure it for surgical planning. It can be measured by obtaining a CT scan of the unaffected side and superimposing it on the affected side [18]; however, radiation exposure is a problem [19]. The linkage simulation avoids scanning of the unaffected side. Flexion and extension of the fingers were dynamically simulated using a predicted rotation axis, allowing accurate osteotomy prediction.

Linkage simulation can simulate finger flexion and extension, and it is not only possible to quantify the degree of deformity, but also to predict the dynamics after reduction. Motion capture is commonly used to analyze upper limb movement [20], but analyses using it cannot measure the rotational deformity caused by malunion because it estimates the joint position indirectly through the skin and is not sufficiently accurate to be used in planning osteotomy surgery. Linkage simulation can reproduce the dynamic flexion and extension of fingers after reduction more accurately than motion capture. Another advantage is that it can be used to treat malunions following multiple finger fractures. It is difficult to assess the rotational angle by visual inspection, and a linkage simulation can reduce the stress on the surgeon.

However, this technique does not perfectly reproduce finger flexion and extension. As has been reported in the past, it is known that finger flexion is not a uniaxial rotational movement but involves translational movement and rotation of the axis itself [11,12]. Therefore, the slight rotation that occurs during joint movements may result in a bias affecting the results. To ensure greater accuracy, one option is to use four-dimensional computed tomography (4D-CT), which captures finger flexion at several positions [21]. However, the degree of rotation is reported to be approximately 2° [11], and we believe that this does not significantly affect the results of malunion reduction using linkage simulation. In addition, considering that in some patients, including the patient we reported previously, reduction was possible almost exactly as in the preoperative simulation, we believe that this simplification is acceptable when considering the burden on the patient due to radiation exposure from 4D imaging.

The limitations of linkage simulation are that, although simplified, the method is complicated, and special software for analysis is required. There is also the possibility that some errors may occur when applying the simulation results to actual surgery. However, as described above, it is possible to reduce the occurrence of errors using a template. Despite the remaining technical limitations, accurate and highly reproducible correction can be achieved with minimal intervention and without the need for repeated reduction and fixation during surgery, regardless of the surgeon.

In the future, automation of some processes may be done in linkage simulation. The introduction of artificial intelligence, such as machine and deep learning, is expected to reduce the burden on surgeons by reducing the amount of manual work and shortening analysis time. The use of convolutional neural networks for bone segmentation has made it possible to reduce the amount of work involved [22]. In addition, there is potential for automation in the determination of the rotation axis, which is the core of this technology, as well as in the creation of the inertial axes and concentric circle models. 

In conclusion, we report on the detailed use of the improved linkage simulation technique in this article and successfully treated nine cases of deformed fractures. Although additional verification is required to confirm the accuracy of the simulation, this method made it possible to quantitatively measure fracture deformity healing.

## Figures and Tables

**Table 1:** Cases treated with linkage simulation.

No.	Age	Side	Malunited Bone	Correction for the Deformity in the Transverse Plane	Degrees of the Deformity in the Transverse Plane	Additional Correction for the Deformity in the Sagittal Plane or Coronal Plane
1	41	R	2-MC	+	25	Coronal plane
2	14	L	5-PP	+	17	
3	53	R	5-MC	+	22	
4	43	R	5-MC	+	14	Sagittal plane
5	34	L	3-PP	+	18	
6	24	R	2-MC	+	7	Sagittal plane
7	64	L	5-PP	+	22	
8	58	L	5-PP	+	8	Sagittal plane
9	10	L	2-PP	+	12	

MC: metacarpal, PP: proximal phalanx.

## Data Availability

The data presented in this study are available on request from the corresponding author. The data are not publicly available due to their containing information that could compromise the privacy of research participants.

## Supplementary Materials

The following supporting information can be downloaded at: https://www.mdpi.com/article/10.3390/diagnostics14161818/s1, Video S1: Linkage simulation. When you install the Grasshopper plug-in with Rhino 7.0 3D-CAD software, interactive finger flexion and extension can be simulated by operating the slide bars.
